# Sensitivity of Nursing Home Cost Comparisons to Method of Dementia Diagnosis Ascertainment

**DOI:** 10.4061/2009/780720

**Published:** 2009-09-22

**Authors:** Ann L. Gruber-Baldini, Bruce Stuart, Ilene H. Zuckerman, Van Doren Hsu, Kenneth S. Boockvar, Sheryl Zimmerman, Steven Kittner, Charlene C. Quinn, J. Richard Hebel, Conrad May, Jay Magaziner

**Affiliations:** ^1^Division of Gerontology, Department of Epidemiology and Preventive Medicine, University of Maryland School of Medicine, Baltimore, MD 21201, USA; ^2^The Peter Lamy Center on Drug Therapy and Aging, Department of Pharmaceutical Health Services Research, School of Pharmacy, University of Maryland, Baltimore, MD 21201, USA; ^3^Department of Pharmaceutical Health Services Research, School of Pharmacy, University of Maryland, Baltimore, MD 21201, USA; ^4^Bronx Veterans Affairs Medical Center, Bronx, NY 10468, USA; ^5^Mount Sinai School of Medicine, New York, NY 10468, USA; ^6^Cecil G. Sheps Center for Health Services Research, School of Social Work, The University of North Carolina, Chapel Hill, NC 27599, USA; ^7^Department of Neurology, University of Maryland School of Medicine, Baltimore, MD 21201, USA; ^8^Department of Medicine, University of Maryland School of Medicine, Baltimore, MD 21201, USA

## Abstract

This study compared the association of differing methods of dementia ascertainment, derived from multiple sources, with nursing home (NH) estimates of prevalence of dementia, length of stay, and costs an understudied issue. Subjects were 2050 new admissions to 59 Maryland NHs, from 1992 to 1995 followed longitudinally for 2 years. Dementia was ascertained at admission from charts, Medicare claims, and expert panel. Overall 59.5% of the sample had some indicator of dementia. The expert panel found a higher prevalence of dementia (48.0%) than chart review (36.9%) or Medicare claims (38.6%). Dementia cases had lower relative average per patient monthly costs, but longer NH length of stay compared to nondementia cases across all methods. The prevalence of dementia varied widely by method of ascertainment, and there was only moderate agreement across methods. However, lower costs for dementia among NH admissions are a robust finding across these methods.

## 1. Introduction

Between 25–74% of all nursing home (NH) residents have Alzheimer's disease or related dementias [1–12]. Estimates of the prevalence of dementia can vary widely, depending on the diagnostic criteria used, source of information for diagnosis, and the population of interest [13]. Studies that use more detailed diagnostic interviews [7] typically find higher prevalence than chart documentation [14].

Just as the prevalence of dementia can vary, estimates of costs from dementia can vary substantially as well. National estimates, all from the 1990s, of the total cost of caring for persons with dementia in the US range from $54 to $120 billion per year [15–18]. Per case dementia cost estimates also vary widely, ranging from $11700 to $174000 per year [17–20]. Although families provide substantial care for individuals with dementia, state and federal governments are responsible for acute medical care and assume a large responsibility for long-term institutional care, primarily through coverage by Medicare and Medicaid. 

One study looking at costs for dementia care for community-dwelling elderly [21] found an incremental cost of dementia of $6927 U.S. dollars per year to Medicare, mostly due to hospitalization; costs for dementia care in long-term care settings are likely to be even higher. In 2000, the total payment to NHs for care was $92.2 billion; of this, 10% came from Medicare ($9 billion) and 48% from Medicaid ($44.3 billion) [22]. Surprisingly, very few studies have focused on the costs of care for NH residents with dementia, despite the fact that 75% of all persons with dementia will eventually reside in an NH [23], and the greatest burden of public expenditures for formal care of those with dementia occurs among those residing in NHs [24]. Those few studies that have looked at NH costs for dementia [21, 25, 26] have failed to examine the differential cost of treating those with dementia, compared to those without dementia, once in a nursing home setting. 

Earlier studies of the costs of dementia have relied heavily on diagnostic information contained in the medical record of NH residents or in the Minimum Data Set (MDS) files. Large national studies, such as the Medical Expenditures Panel Study (MEPS) [12], which base findings on the MDS, underestimate rates of dementia by as much as 30% [14]. Others, such as the National Medical Expenditures Study (NMES) [15] relied on nurses' reports of chart-based diagnoses. These, too, have limitations in that they are not obtained by any consistent diagnostic standard and underestimate the true prevalence of dementia [27]. Smaller studies of costs have been able to use other methods, which include mental status testing [16, 17, 19, 23], but these studies are limited because low functioning on mental status tests is only one aspect of a dementia diagnosis [27, 28]. None of these studies have directly compared costs of dementia versus non-dementia cases.

In addition to charts and mental testing, diagnostic information from Medicare claims has also been used to examine cost difference between those with and without dementia in community settings. However, Medicare data may also greatly underestimate the prevalence of dementia and provide biased estimates of the impact of dementia. Two studies (not in NHs) have compared data from Medicare claims files within a group of community-dwelling elderly with dementia. Both studies have found undercounts of dementia cases ranging from 20% [29] to 87% [30] among patients with known Alzheimer's disease. A more recent study [31] explored the correspondence of Medicare claims of dementia to survey data and brief cognitive screening in 5089 community-dwelling elderly. They also found that 5 years of Medicare claims captured the largest number of dementia cases (562/855 = 65.7%), but that there was substantial nonoverlap in diagnoses (kappa between Medicare and survey report = 0.41, 76% agreement). 

No research, to our knowledge, has examined the correspondence of the dementia diagnosis in the NH chart with prior Medicare claims, and none has compared claims and charts to a “gold standard” dementia diagnosis, such as an expert panel. Therefore, this study will compare dementia diagnosis derived from Medicare data in the year prior to NH admission with both NH chart data and with a dementia diagnosis ascertained by an expert panel. This expert panel included neurologists, psychiatrists, and a geriatrician, who followed a standardized protocol and used DSM-III-R criteria for a diagnosis [14]. Differences in the prevalence, characteristics of dementia cases at baseline, and Medicare costs per month while in the initial NH are examined by these alternate sources of dementia diagnosis. By doing so, this study will permit comparison of the effect of different approaches in estimating the prevalence of dementia on cost estimates of the disease. 

This study relies on data from an existing epidemiologic nursing home admissions cohort [14, 32]. The original study was designed with the hypotheses that (1) there is a large under-recognition of dementia in NHs and hence the reliance on the panel methodology to determine cases in the study, and (2) that residents with dementia entering a nursing home would die at a higher rate and develop more adverse health events than residents without dementia. Underlying this second hypothesis was the fact that dementia can reduce capacity to perceive and report early symptoms, participate in treatment decisions, and adhere to treatment recommendations [33, 34]. Consequently, nursing home staff may be less able to intervene early [34], as care may be provided only after observing severe symptoms [34, 35]. This delay may result in more serious medical problems, more extensive treatment, and increased mortality. Our previous work [14, 32] has not supported this hypothesis on mortality, adverse medical events, or Medicare costs using a panel determination of dementia, so we wanted to test whether the source of the dementia determination would impact our findings. It would be possible that cases that are recognized by nursing home staff to have dementia (chart-documented cases) may be more severe than those unrecognized by staff (research panel cases), leading to communication and adherence difficulties, and/or that the recognition of dementia creates more potential treatment bias based on negative perceptions about residents with dementia.

## 2. Design and Methods

### 2.1. Sample

The sample for this analysis was 2050 residents, ages 65 and older, newly admitted between September 1992 and March 1995 to 59 Maryland NHs who had complete chart abstractions (capturing the first 2 weeks of the NH stay) and Medicare claims (for 360 days preceding admission through 30 days afterwards). The 59 NHs were randomly selected from 221 NHs in the state and recruited within each of 15 strata, defined by five regions and bed size (<50, 51–150, > 150), such that the proportion of beds represented for each stratum was approximately the same as that stratum's proportion of all beds statewide. Sixty-four facilities were eventually contacted to participate in the study. Four (6%) refused to participate, and one facility that agreed to participate had no new admissions over the course of the study. A more detailed description on the methods of the parent study may be found elsewhere [14].

The overall sample of the parent study included 2285 residents at baseline, from a total of 3283 eligible subjects, of whom 30% refused participation [14]. Residents age 65 years and older who had not resided in a nursing facility or chronic care facility for 8 or more days in the previous year were eligible. All participants were followed until discharge, death, or for up to two years after initial enrollment. The protocol for the initial study, chart follow-up, and the additional of Medicare claims was approved by the Institutional Review Board of the University of Maryland Baltimore. Of the 2285 residents in the study, 2050 (89.7%) were matched with Medicare claims records and are included in these analyses. More detail on the methods of examining Medicare costs in this sample is available in a previously published paper [32].

### 2.2. Measures

#### 2.2.1. Dementia Determination

*Panel dementia status* was determined in accordance with Diagnostic and Statistical Manual III-R criteria [36] by an expert panel of geriatric psychiatrists, neurologists, and a geriatrician. A detailed description of the dementia ascertainment methodology is found elsewhere [27]. Briefly, two panelists (one neurologist and one psychiatrist) rendered independent diagnoses in three categories: dementia, no dementia, or difficult to diagnose. If they agreed, that was the determination. When there was disagreement (*n* = 526), the case was brought to a full panel discussion with 2 neurologists, 2 psychiatrists, and a geriatrician. The geriatrician convened the conference and adjudicated the final diagnosis. The cases classified as “difficult to diagnose” were added to the “no dementia” group for purposes of this report. Information used for dementia diagnosis included previous and current cognitive and functional status, in addition to demographic characteristics and affective, social, and behavioral status [14].

*Chart diagnoses of dementia* (presence versus absence) was coded from NH chart records (including nursing note, physician orders, admission sheets, medication orders, but excluding the MDS) from the first two weeks of the NH stay. Complete copies of the baseline NH chart were photocopied and coded by a research abstractor for diagnosis of Alzheimer's disease or other dementias. Multiple dementia etiologies could be noted. Other mentions of cognitive/memory impairment and senility in the first two weeks were noted separately and coded, but not included in the dementia category for these analyses. Charts were available for 2273 of the 2285 residents (99.5%) at baseline. 

*Designation of dementia from the Minimum Data Set* (MDS) was also available for most subjects (86% of baseline). This measure was obtained from a checklist of medical conditions on the MDS, including any indication of Alzheimer's disease or other dementias. Previous work in this sample [37] has shown that the MDS dementia coding in this sample corresponded highly with chart diagnosis (89% agreement, kappa = 0.78). Thus, the MDS diagnosis was not included in further analyses (as it is fairly redundant with the chart information).

*Medicare diagnosis of dementia* was obtained from any indication using ICD-9-CM codes 290.0-3 or 290.9 (senile, presenile, and degenerative dementia), 290.4 (vascular/arteriosclerotic dementia), 294–294.9 (amnestic syndrome, organic brain syndrome, or dementia listed in other conditions), and/or 331–331.9 Alzheimer's Disease or cerebral degeneration (there were no codes for 331.1 Pick's disease). Codes were associated with all the Medicare claims (Part A and Part B) in the 360 days preceding NH admission or during the month of admission. The coding system combined codes from Martin et al. [26] and Taylor et al. [30] for dementia. Acute delirium and “dementias specifically associated with (as a result of) other diseases” (as coded in ICD-9-CM) were not included. Medicare claims allow one principal ICD-9-CM diagnosis code to be submitted with a claim; Medicare allowed up to 14 additional codes during the time frame we used (1992–1995). Diagnosis codes came from forms UB92 and HCFA 1500; all claims have one of these forms. Thus, if a diagnosis is reported, it should have been captured. There were a total of 1 097 313 Medicare records used to capture these diagnoses, of which 30 349 (2.8%) had any diagnosis of dementia.

We initially included ICD code “797” (senility without mention of psychosis) per the work of others [26, 30, 38]. However, this code that was found on only 0.8% of claims and in only 1.8% of our sample had little impact on prevalence, but slightly decreased specificity. It was subsequently dropped from the coding of Medicare dementia and not analyzed further.

#### 2.2.2. Other Measures of Cognitive Function

The *Mini-mental State Examination* (MMSE) [39] is a 30-point scale, with scores of 23 or less indicative of some cognitive impairment and scores of 17 or less indicative of moderate-to-severe impairment. Subjects who were comatose at the time of MMSE interview received a score of zero. The MMSE was available for 1264 subjects (62%) of the 2050 subjects at baseline.

The *MDS-Cognition Scale (MDS-COGS)* [40] is a cognitive scale derived from the MDS. The MDS-COGS has an 11-point scale which ranges from 0 = cognitively intact to 10 = very severe impairment based on care provider ratings. Items include short-term memory, long-term memory, three orientation items, cognitive skills for daily decision-making, being understood by others, and self-performance in dressing. The MDS-COGS was available for 1718 subjects (84%) of the 2050 subjects at baseline.

#### 2.2.3. Two-Year Disposition Measures

For two years following the date of NH admission, length of stay (LOS), discharge disposition (remained in the NH, discharged to community, or discharged to another NH,), and death were determined from a two-year medical record review. Subsequent periods of hospitalization or rehabilitation were considered part of the initial NH stay.

#### 2.2.4. Merged Cost Data Measures

Medicare and Medicaid data were available from 1992–1997, which permitted data for at least 9 months before NH admission for all participants, and up to 6 years postadmission for some participants. For this analysis, we chose to study up to two years postadmission, since this was the time frame that we could verify that patients remained in the initial NH. Based on Medicare claims, measures of cost and utilization of care were developed. The data set aggregated subjects' cost and utilization information contained in the claims records on a per-person-month (ppm) basis by service type and payer. The date of nursing home admission was the reference date (and was included in the first month of NH stay).

#### 2.2.5. Covariates

Baseline data were collected by trained lay interviewers from multiple sources including structured interviews with residents, nursing staff, and significant others (the designated family member, friend, or legal guardian who could provide consent and/or knew the resident best). Baseline functional status was assessed with a modified version of the Katz *Activities of Daily living (ADL) Scale*, which measures ability in bathing, dressing, toileting, transferring, feeding, and continence [41]. Each of 6 ADL domains was asked of a nursing staff member and scored as dependent or fully independent; a summary measure indicated the number of domains in which residents were dependent. *Chairfast* and *bedfast*, were whether a resident was confined to a chair or bed, were based on information provided from the MDS or nursing staff interviews; these two variables were included separately in the model in addition to the number of ADLs not fully independent. 

*Age, gender, race, education*, and *marital status* were obtained from significant-other interviews or from charts if not available from significant others. Martial status was dichotomized as married versus other (widowed, divorced, and never married). Race was dichotomized as white, non-Hispanic versus other. 

The *Resource Utilization Group Score-Case Mix Index (RUGS CMI)* is derived from the MDS forms at NH admission. Since our data preceded version 2 of the MDS, we used the original RUG-III classification and weighting derived by Fries and colleagues [42]. A hierarchical grouping of 7 overall classifications with 44 groupings was created from the MDS (ranging from high utilization groups such as specialized rehabilitation to low utilization groups such as reduced physical functioning) and a relative nursing unit weighting was applied. The score is representative of amount of skilled nursing care required by the resident, with all weights relative to 1. The RUGS CMI has been used as the basis of Medicare NH prospective payment to skilled nursing facilities since late 1998 [43, 44]. 

*Medicare qualified SNF stays* (which require a hospital admission prior to entry) were inferred from any SNF payment from Medicare that included the index admission date. Residents were considered *Medicaid eligible* at admission if they had any month with Medicaid eligibility in the 12 months prior to admission or within 30 days of NH admission.

The *Diagnostic Cost Group/Hierarchical Coexisting Condition (DCG/HCC)*, a risk adjuster derived from Medicare claims, was used to correct for expected costs based on utilization prior to NH admission. The DCG/HCC is the basis for the “selected significant disease model” that the Center for Medicare and Medicaid Services (CMS) has used to set capitation rates for Medicare HMOs since January, 2004 [45]. Our application of the DCG/HCC model used the 12 months of preadmission Medicare claims to create a predicted value for Medicare services in the immediate post-admission year for each study subject. Because our interest is in finding the marginal contribution of dementia to residents' cost patterns, we explicitly excluded the HCC category that represents dementias (HCC 49).

### 2.3. Data Analyses

Three classifications of dementia (panel, medical chart, and Medicare claims) were examined to determine if prevalence and estimates of the cost of care differ by the ascertainment method. Comparison of agreement across the methods of ascertainment was calculated using Cohen's kappa statistic, which measures the proportion of agreement beyond the amount expected by chance alone. Guidelines for interpreting Kappa statistics suggest that values between 0.81–1.00 indicate almost perfect agreement, 0.61–0.80 substantial agreement, 0.41–0.60 moderate agreement, 0.21–0.40 fair agreement, and values less than 0.21 are poor or slight agreement [46].

Using the panel's determination as the criterion variable “gold standard,” false positive and negatives rates were determined for each ascertainment method, and hence sensitivity and specificity. Mean costs were determined separately for the true positives, false positives, true negatives, and false negatives. Post-hoc t-tests within ANOVA were used to evaluate the equality of the means between preplanned groups (cases missed versus captured when comparing methods of ascertainment).

Predictions of cost ratios of dementia versus non-dementia residents under different methods of ascertainment were estimated. All costs were converted to 1997 current dollar values to adjust for the effect of inflation over time using the Consumer Price Index (CPI) with December 1997 as the base period. 

Intrafacility correlations could lead to underestimates for the standard errors of regression coefficients. A technique described by Huber and White [47, 48] and expanded on by others to include correlated cases [49, 50] was used to account for cluster sampling and provides robust variance estimates. This technique makes use of a grouping variable, in this case facility, without requiring its inclusion in the regression model. It uses a theoretical bootstrap method for correcting the standard errors of the regression coefficients and can be applied in regression analyses using many distributions (including gamma). Predictions of cost ratios of dementia versus non-dementia residents were estimated in STATA using a generalized linear model (xtgee) with a log-link and a gamma distribution, utilizing a robust variance estimate that adjusts for within-cluster (NH facility) correlation [51]. We chose a gamma distribution as this approach has gained acceptance as the preferred distribution for costs [52], as costs were right skewed and included numerous zero values. Predictions of NH length of stay by different ascertainment models were also estimated using a generalized linear model, but with a Gaussian distribution assumption.

## 3. Results

### 3.1. Prevalence of Dementia under Different Methods

Table 1 (1st data column) provides the prevalence of dementia from the three ascertainment methods. The expert panel found the highest prevalence of dementia, in 48% of the sample. The rate from Medicare was 39% and from the medical chart 37%. Overall, 60% of the sample had some indicator of dementia, with similar prevalence rates from panel as from a combined chart plus Medicare claims (48%. versus 49%).

For charts and Medicare claims, the most common “subtype” of dementia noted was senile dementia (data not shown), which was found in 29.8% of the sample by Medicare claims, and 22.9% of the sample by chart. Alzheimer's disease was found in 18.1% of the sample by Medicare claims, and 14.6% of the sample by chart. Vascular dementia was found in 7.7% of the sample by Medicare claims and in 3.4% of the sample by chart.

### 3.2. Agreement between Methods

Table 1 also presents the concordance between the methods of ascertainment. Agreement between methods was moderate (72–78%; kappas 0.43–0.55), with the highest concordance between NH chart and the panel. The use of a combined chart and Medicare claim determination had the highest sensitivity (77%) with the panel determination, while the chart alone had the highest specificity (89%).

### 3.3. Differences in Dementia Cases by Ascertainment Method

Table 2 presents the differences in dementia cases “captured” versus “missed” by each ascertainment method. The “all methods” cases looked at dementia and non-dementia by combining all three methods; if any method noted dementia it was considered a dementia case. Significant baseline differences were found for age, ADL, chairfast, bedfast, DCG/HCC, RUGS CMI, Medicare qualified stay, MMSE, and MDSCOGS. Significant differences were also found for NH length of stay, death, and discharge home. Panel dementia cases missed by claims or chart were significantly older age, more ADL impaired, more chairfast, less bedfast, more impaired on MMSE and MDSCOGS, had longer NH length of stay, were less likely to die in first 90 days, and were less likely to be discharged home. Dementia cases captured by Medicare had the highest DCQ/HCC (predicted utilization), were more likely admitted as a Medicare postacute qualified stay, and were the least impaired on the MMSE. Chart dementia cases captured had the lowest RUGS CMI index (indicating less need for skilled care).

### 3.4. Cost Ratios and NH Length of Stay by Method of Ascertainment

Table 3 shows the average PPM Medicare costs after admission and NH length of stay differences by the three dementia ascertainment methods. Estimated Medicare costs per month were lower for those with dementia compared to those without dementia under all ascertainment methods. The ratios for dementia/non-dementia costs varied by method, such that the panel and chart estimated relative costs ppm of 0.61-0.62, while the claims estimated dementia cost ppm was 0.77. Adjustment for covariates to account for demographics, functional impairment, DCG/HCC predicted costs, and qualification for Medicaid or Medicare postacute stay did bring the ratios slightly closer to 1.00 (0.65–0.80), but did not eliminate the statistically significant lower costs for dementia cases. In contrast, dementia cases had significantly higher overall NH LOS under all methods. Panel dementia cases had the highest overall NH length of stay.

### 3.5. Differences by Ascertainment Method in Medicare Cost Categories

Table 4 shows the impact of dementia by ascertainment method across the Medicare cost categories. In general, all the cost ratios are lower than 1.0, which indicates lower costs for dementia across all ascertainment methods and cost categories. The “other” cost category (which includes outpatient, home health, durable medical equipment, and hospice) has the lowest costs for dementia relative to non-dementia, and this magnitude is similar across all the ascertainment methods. Inpatient hospitalization costs show the greatest differences in magnitude by ascertainment methods, such that a diagnosis of dementia ascertained by Medicare claims estimated similar inpatient hospital costs among residents with and without dementia, whereas other ascertainment methods showed lower inpatient hospital costs among residents with dementia.

### 3.6. Medicare Costs by Medicare Qualified SNF Stay

Table 5 examines whether the impact of dementia ascertainment method is different by whether the resident enters under a Medicare Qualifying (postacute) stay (in which NH care is reimbursed by Medicare). The first 90 days is also examined separately, since Medicare directly reimburses SNF stays during that time period. Similar patterns are seen for PPM costs over the whole 2-year NH stay and in the first 90 days. Overall, the impact of dementia on lower Medicare costs is greater for those not entering under a qualifying stay; ratios for those entering under a qualified stay are close to 1.0. There is much consistency across methods, although the Medicare claims diagnosis of dementia does provide a more similar cost ratio (closer to 1.0) under a nonqualifying stay than other methods.

## 4. Discussion

Previous work from our NH cohort found that costs for residents with dementia are lower than those without dementia, and that adjustment for resident characteristics and costs prior to admission cannot account for these cost differences [32]. This paper extends our work on the new admissions cohort study, by providing direct comparisons of multiple measures of dementia, including the Medicare records and NH chart, and examining the impact of these differing ascertainment methods on estimated costs. This potentially allows a comparison of findings from our study to community sample data (which heavily rely on Medicare claims for dementia determination) and to NH national datasets (which rely heavily on NH chart and/or MDS). The results from this paper demonstrate that the lower costs for dementia among NH admissions is a robust finding across dementia ascertainment methods. 

While the cost ratios were similar, there was, however, only moderate agreement across methods of dementia ascertainment. The differences in overall prevalence are important in determining the overall cost (versus relative cost) of dementia care in NHs. The panel found a higher prevalence of dementia than any other method of ascertainment; panel dementia cases had lower relative average PPM costs, but longer NH length of stay, compared to non-dementia cases. Medicare claims appear to capture dementia patients with higher Medicare utilization (DCG/HCC and qualified stay), as might be expected since the capture of diagnosis is dependent on having claims. Panel-detected dementia cases were significantly older age, more ADL impaired, more chairfast, less bedfast, less cognitively impaired, had longer NH length of stay, less likely to die in first 90 days, and less likely to be discharged home than those detected by other methods. This prediction provides additional validity to the use of our panel process, in that the panel dementia cases were more likely the long-stay nursing home cases. Chart dementia cases captured were the least likely to come in under a Medicare qualified stay and had the lowest RUGS CMI index, perhaps indicating less need for skilled care. 

One implication of the greater prevalence of dementia found by panel determination and difference in the casemix is that expert or specialist screening for a diagnosis of dementia on admission to the nursing home might help nursing home staff to deliver more patient-centered care and to weigh the potential benefits and harms of health interventions. However, such an approach would require significant resources and would need to be tested. 

Data for this paper were derived using a NH admission cohort and thus represent the highest cost dementia cases. Persons who enter a nursing home are, by definition, more physically sick and/or functionally disabled than those who remain in the community. This might explain why our costs for those with dementia are low relative to non-dementia, since many of the non-dementia cases are also very ill (as shown by the higher DCG/HCC means for those without dementia) and most of our subjects enter the nursing home through a hospitalization and related postacute qualifying stay (67% of non-dementia and 46% of dementia cases). Much previous work has found that most of the higher cost for dementia care in the community is due to hospitalization costs [21]. Our previous work has also shown that the lower costs to NHs for people with dementia are not seen when looking only at those admitted for a Medicare Qualifying Skilled Nursing Facility, in which both groups may be more similar on severity of comorbidities [32].

Although there are potential differences between the groups in underlying medical illness, we cannot rule out the alternative hypothesis that the lower costs for people with dementia are due to lower attention and treatment because of either inability of people with dementia to verbalize their care needs or biases towards people with dementia. Certainly, that was our underlying hypothesis in designing the overall study, although we expected that the lack of treatment would lead to higher rates of other adverse events (e.g., deaths, serous infections, and hospitalizations), which has not been seen in our data [14]. Furthermore, we would have expected that this difference to be more pronounced in “recognized” dementia cases versus those determined from our panel, and none of our results has shown that the method of determination affects utilization or outcomes. 

Those who enter NHs are more likely to have many Medicare claims in the year prior to admission, minimizing the potential selection bias of people not needing/seeking care being missed. While Taylor et al. [30] recommend at least 3 years of Medicare data to capture dementia diagnoses, our data had only 13 months of Medicare claims. However, the period pre-NH admission is a high intensity medical use time, so it is less likely that we lacked medical visits in examining dementia codes. 

We used the panel determination of dementia as the major criterion measure of dementia in our study; however our diagnosis is certainly not a “gold standard.” Previous reports from our group have shown this determination to be as valid as a clinician assessment [27]; however, any dementia diagnosis (relying on secondary information and not direct clinical examination) may not be totally accurate. 

It should be considered that the data for this study was derived from 59 NHs in a single state (Maryland) and was conducted in 1992–1997, which is before the implementation of the Medicare NH Prospective Payment System (PPS) in the US. The NH-PPS has more tightly linked the reimbursement for postacute skilled care (which the majority of the sample was admitted under) to the level of rehabilitation care provided and the medical needs of the patient. While dementia and related ADL impairment are a part of the current reimbursement hierarchy, the level of reimbursement for solely dementia cases is relatively low and remains so even in the newest refinement of the RUGS [53]. The expectation of relatively lower costs for those with dementia as their primary reason for NH care was based on work done on nursing time [42], the findings of which are in line with the lower relative Medicare costs we found. However, our previous findings also showed that dementia cases are more similar in costs to non-dementia cases if they entered for a Medicare Qualified SNF stay [32].

Our cost data was estimated from 1992 to 1997, and we standardized the absolute cost amounts to December 1997 dollars. Certainly these estimates would be much higher in 2009, given that the Consumer Price Index has risen 133.7% from December 1997 to June 2009 [54]. However, most of our data is presented as relative rates, which should not be impacted by inflation. Relatedly, as noted in the introduction, most cost reports of the caring for people with dementia date to the early 1990s and more current estimates of these costs would be valuable for researchers and policy analysts.

Since the time of the study, the use of assisted living facilities for care, especially for dementia, has increased [22]. Many assisted living facilities specialize in care for residents with mild or moderate dementia. It is likely that many of the mild low-cost dementia cases included in our sample (and more likely to be picked up by the panel) may now have care provided in these alternative settings, which might bring the cost ratios for dementia versus non-dementia closer to 1. The finding of more similar cost ratios for between dementia and non-dementia cases admitted as a postacute qualifying stay might be more appropriate to this post-PPS care environment, but that remains an empirical question to be answered by other data.

Also, there may have been changes over time in attention and coding for dementia in Medicare claims records since 1992 [55, 56]. In September 2001, CMS issued a special directive due to concerns about the limiting of certain claims for persons with dementia [57]. Reimbursement for many claims during the time period we studied did not require secondary diagnoses to be listed, so there is certainly concern that the Medicare claims undercount dementia cases.

One finding that was consistent across ascertainment methods is that dementia patients have longer lengths of NH stay. Thus, while dementia cases have lower per month costs, their increased length of stay translates to similar costs per total episode of NH care. Policy makers and managed care plans should consider this tradeoff when determining how to set rates for dementia cases.

In conclusion, we found that most readily available ascertainment methods for diagnosing dementia in NHs vastly undercount the number of dementia cases admitted to nursing homes and may miss those cases most likely to remain the longest in the NH environment. However, the per month costs while in the NH were consistently lower among dementia cases, as compared to non-dementia admissions, across all methods of dementia ascertainment. Future research should verify that this pattern remains given the growth of alternate long-term care settings and changes in Medicare reimbursement.

## Figures and Tables

**Table 1:** Prevalence and agreement between methods of dementia diagnosis (*n* = 2050).

	Overall	Correspondence with panel dementia				Correspondence with chart dementia			
	prevalence	Agreement	Kappa	Sensitivity	Specificity	Agreement	Kappa	Sensitivity	Specificity
Panel	48.0%	—	—	—	—	78%	0.55	65%	89%
Chart	36.9%	78%	0.55	65%	89%	—	—	—	—
Medicare claims	38.6%	72%	0.43	61%	82%	78%	0.54	73%	82%
Chart or Medicare	48.6%	78%	0.55	77%	78%	—	—	—	—
Panel or chart or Medicare	59.5%	—	—	—	—	—	—	—	—

**Table 2:** Resident characteristics of dementia cases, and significance of differences, by ascertainment method.

	Mean or percent								Significance of differences		
	No dementia	Dementia cases							Comparisons of dementia		
									missed by method+		

	Any method	Any method	Panel captures	Panel missed	Chart captures	Chart missed	Medicare captures	Medicare missed	Panel versus chart	Panel versus Medicare	Chart versus Medicare

	*n* = 830	*n* = 1220	*n* = 984	*n* = 236	*n* = 757	*n* = 463	*n* = 792	*n* = 428			

*Baseline (NH entry)*											

Age (mean)	80.3	82.4	82.9	80.6	82.5	82.3	82.2	82.9	∗	∗∗∗	
Education (mean)	11.3	10.3	10.3	10.1	10.3	10.3	10.1	10.6			
Gender % male	24.8	31.0	30.1	34.7	29.7	33.0	31.4	30.1			
Race % nonwhite	12.8	22.1	22.7	19.9	22.2	22.0	23.1	20.0			
Currently Married %	19.9	26.7	27.2	24.6	28.4	24.0	27.8	24.8			
Katz ADL (mean)	3.1	4.3	4.4	3.5	4.2	4.2	4.3	4.1	∗∗∗	∗∗	
Comorbidity (mean)	2.7	2.6	2.6	2.7	2.4	2.9	2.5	2.8			
Chairfast %	27.0	30.8	31.5	27.4	27.3	36.5	29.3	33.6	∗∗		
Bedfast %	9.3	7.6	5.9	15.2	6.7	9.1	8.0	7.0	∗∗	∗∗∗	
DCG/HCC (mean)	14,757.1	13,250.2	12,142.5	17,706.9	11,779.8	15,569.3	13,522.4	12,708.8		∗∗∗	∗∗∗
RUGS CMI (mean)	1.09	1.09	1.09	1.13	1.04	1.20	1.09	1.12	∗		∗∗∗
Medicaid at admission %	17.8	27.4	27.9	25.0	25.9	29.8	27.8	26.6			
Medicare qualified stay %	67.0	45.7	43.9	55.9	40.7	55.1	46.6	46.0		∗∗	∗∗∗
MMSE (mean)	24.4	13.7	12.4	22.1	12.3	15.7	13.0	14.9	∗∗∗	∗∗∗	∗
MDSCOGS (mean)	1.5	4.9	5.2	3.4	5.5	3.9	5.4	4.0		∗	

*NH disposition*											

NH length of stay (mean)	204.8	375.8	402.4	265.0	398.4	338.5	380.1	367.9	∗∗	∗∗∗	∗
Died first 90 days %	15.3	12.1	9.7	22.0	11.7	12.8	12.4	11.5	∗∗∗	∗∗∗	
Discharge home (2 years) %	48.5	18.3	15.6	29.3	17.2	20.1	17.7	19.4	∗∗	∗∗	

+Significance of preplanned *t*-test comparisons of missed dementia cases across methods (dummy coefficient from regression analyses).

***p < .001, **p < .01, *p < .05.

**Table 3:** Dementia versus nondementia nursing home cost (per patient month) and length of stay ratios by method of ascertainment.

Ratios: dementia versus non-dementia			
	Costs PPM^a^	Costs PPM^a^	NH LOS
	Raw (95% C.I.)	adjusted (95% C.I.)^b^	Raw (95% C.I.)^c^
Panel	0.61 (0.53, 0.70)	0.65 (0.52, 0.80)	1.55 (1.40, 1.72)
Chart	0.62 (0.54, 0.72)	0.70 (0.58, 0.86)	1.38 (1.24, 1.52)
Medicare	0.77 (0.66, 0.90)	0.80 (0.67, 0.96)	1.29 (1.18, 1.40)
Chart or medicare	0.70 (0.62, 0.80)	0.78 (0.66, 0.91)	1.38 (1.25, 1.52)
Chart or medicare or panel	0.66 (0.59, 0.74)	0.68 (0.57, 0.81)	1.53 (1.38, 1.70)

^a^Costs inflated to 1997 dollars, models from GEE assuming Gamma distribution for costs. Costs are those incurred in the nursing home up to 24 months after admission and only include those periods in which the patient was in the initial nursing home.

^b^Adjusted for age, gender, race, marital, ADL, chairfast, bedfast, DCG/HCC, Medicare qualified stay, and Medicaid status at admission.

^c^Models from GEE assuming Gaussian distribution for length of stay (LOS).

**Table 4:** Dementia versus nondementia cost ratios for different Medicare cost categories by method of ascertainment.

Adjusted cost ratios PPM (95% confidence intervals)^a^ : dementia versus non-dementia					
	Overall	SNF	Inpatient Hospital	Physician	Other services^b^
Panel	0.65 (0.52, 0.80)	0.68 (0.43, 1.09)	0.75 (0.50, 1.12)	0.74 (0.63, 0.88)	0.52 (0.43, 0.62)
Chart	0.70 (0.58, 0.86)	0.65 (0.44, 0.98)	0.78 (0.56, 1.09)	0.82 (0.72, 0.93)	0.46 (0.36, 0.59)
Medicare	0.80 (0.67, 0.96)	0.69 (0.49, 0.99)	0.96 (0.71, 1.29)	0.85 (0.73, 1.00)	0.54 (0.45, 0.65)
Chart or Medicare	0.78 (0.66, 0.91)	0.64 (0.45, 0.90)	0.94 (0.74, 1.20)	0.86 (0.77, 0.97)	0.51 (0.43, 0.61)
Chart or Medicare or Panel	0.68 (0.57, 0.81)	0.61 (0.40, 0.95)	0.81 (0.60, 1.10)	0.78 (0.68, 0.90)	0.50 (0.43, 0.59)

^a^Costs inflated to 1997 dollars, models from GEE assuming Gamma distribution. Costs are those incurred in the nursing home up to 24 months after admission and only include those periods in which the patient was in the initial nursing home. Cost ratios are adjusted for age, gender, race, marital status, ADL, chairfast, bedfast, DCG/HCC, Medicare qualified stay, and Medicaid status at admission.

^b^Other services include outpatient, home health, durable medical equipment, and hospice costs.

**Table 5:** Dementia versus nondementia cost ratios by method of ascertainment by whether admitted to NH under a Medicare qualifying stay (MQS).

Adjusted cost ratios (95% confidence intervals)^a^ : dementia versus non-dementia				
	Overall Costs PPM		Costs in the first 90 days	
	MQS	Not MQS	MQS	Not MQS
Panel	0.95 (0.82, 1.09)	0.43 (0.30, 0.60)	0.99 (0.89, 1.11)	0.51 (0.34, 0.74)
Chart	0.93 (0.81, 1.08)	0.50 (0.37, 0.68)	0.94 (0.84, 1.06)	0.52 (0.39, 0.68)
Medicare	0.94 (0.78, 1.12)	0.68 (0.48, 0.97)	0.96 (0.83, 1.10)	0.65 (0.49, 0.87)
Chart or Medicare	0.99 (0.88, 1.12)	0.55 (0.41, 0.75)	0.99 (0.90, 1.09)	0.57 (0.44. 0.73)
Chart or Medicare or Panel	0.93 (0.82, 1.04)	0.47 (0.32, 0.67)	0.97 (0.88, 1.06)	0.55 (0.40, 0.75)

^a^Costs inflated to 1997 dollars, models from GEE assuming Gamma distribution. Costs are those incurred in the nursing home up to 24 months after admission and only include those periods in which the patient was in the initial nursing home. Models adjusted for age, gender, race, marital, ADL, chairfast, bedfast, DCG/HCC, and Medicaid status at admission.

## References

[1] Lair TJ, Lefkowitz DC (1990). *Mental Health and Functional Status of Residents in Nursing and Personal Care Homes*.

[2] Hing E, Sekscenski E, Strahan G (1989). *The National Nursing Home Survey: 1985 Summary for the United States*.

[3] Magaziner J, Zimmerman SI, Fox KM, Burns BJ (1998). Dementia in United States nursing homes: descriptive epidemiology and implications for long-term residential care. *Aging and Mental Health*.

[4] Garrard J, Buchanan JL, Ratner ER (1993). Differences between nursing home admissions and residents. *Journal of Gerontology*.

[5] Rovner BW, Kafonek S, Filipp L, Lucas MJ, Folstein MF (1986). Prevalence of mental illness in a community nursing home. *American Journal of Psychiatry*.

[6] Canadian Study of Health and Aging Working Group (1994). Canadian study of health and aging: study methods and prevalence of dementia. *Canadian Medical Association Journal*.

[7] Rovner BW, German PS, Broadhead J (1990). The prevalence and management of dementia and other psychiatric disorders in nursing homes. *International Psychogeriatrics*.

[8] Lewis MA, Leake B, Clark V, Leal-Sotelo M (1989). Case mix and outcomes of nursing home patients. The importance of prior nursing home care and admission from home versus hospital. *Medical Care*.

[9] Burns BJ, Larson DB, Goldstrom ID (1988). Mental disorder among nursing home patients: preliminary findings from the National Nursing Home Survey pretest. *International Journal of Geriatric Psychiatry*.

[10] Engle VF, Graney MJ (1993). Stability and improvement of health after nursing home admission. *Journals of Gerontology*.

[11] Bernabei R, Gambassi G, Lapane K (1999). Characteristics of the SAGE database: a new resource for research on outcomes in long-term care. SAGE (Systematic Assessment of Geriatric drug use via Epidemiology) Study Group. *Journals of Gerontology. Series A*.

[12] Krauss NA, Altman BM (1998). *Characteristics of Nursing Home Residents—1996*.

[13] Erkinjuntti T,  Østbye T, Steenhuis R, Hachinski V (1997). The effect of different diagnostic criteria on the prevalence of dementia. *The New England Journal of Medicine*.

[14] Magaziner J, German P, Zimmerman SI (2000). The prevalence of dementia in a statewide sample of new nursing home admissions aged 65 and older: diagnosis by expert panel. *Gerontologist*.

[15] Short P, Feinleib S, Cunningham P (1994). *Expenditures and Sources of Payment for Persons in Nursing and Personal Care Homes*.

[16] Leon J, Cheng C-K, Neumann PJ (1998). Alzheimer’s disease care: costs and potential savings. *Health Affairs*.

[17] Rice DP, Fox PJ, Max W (1993). The economic burden of Alzheimer’s disease care. *Health Affairs*.

[18] Ernst RL, Hay JW (1994). The US economic and social costs of Alzheimer’s disease revisited. *American Journal of Public Health*.

[19] Hu T, Huang L, Cartwright WS (1986). Evaluation of the costs of caring for the senile demented elderly: a pilot study. *Gerontologist*.

[20] Huang L-F, Cartwright WS, Hu T-W (1988). The economic cost of senile dementia in the United States, 1985. *Public Health Reports*.

[21] Bynum JPW, Rabins PV, Weller W, Niefeld M, Anderson GF, Wu AW (2004). The relationship between a dementia diagnosis, chronic illness, Medicare expenditures, and hospital use. *Journal of the American Geriatrics Society*.

[22] Centers for Medicare and Medicaid Services http://www.cms.hhs.gov/TheChartSeries/02_CMS_Facts_Figures.asp.

[23] Welch HG, Walsh JS, Larson EB (1992). The cost of institutional care in Alzheimer’s disease: nursing home and hospital use in a prospective cohort. *Journal of the American Geriatrics Society*.

[24] Hay JW, Ernst RL (1987). The economic costs of Alzheimer’s disease. *American Journal of Public Health*.

[25] Taylor DH, Sloan FA (2000). How much do persons with Alzheimer’s disease cost Medicare?. *Journal of the American Geriatrics Society*.

[26] Martin BC, Ricci JF, Kotzan JA, Lang K, Menzin J (2000). The net cost of Alzheimer disease and related dementia: a population-based study of Georgia Medicaid recipients. *Alzheimer Disease and Associated Disorders*.

[27] Magaziner J, Zimmerman SI, German PS (1996). Ascertaining dementia by expert panel in epidemiologic studies of nursing home residents. *Annals of Epidemiology*.

[28] American Psychiatric Associations, Committee on Nomenclature and Statistics (1995). *Diagnostic and Statistical Manual of Mental Disorders*.

[29] Newcomer R, Clay T, Luxenberg JS, Miller RH (1999). Misclassification and selection bias when identifying Alzheimer’s disease solely from Medicare claims records. *Journal of the American Geriatrics Society*.

[30] Taylor DH, Fillenbaum GG, Ezell ME (2002). The accuracy of Medicare claims data in identifying Alzheimer’s disease. *Journal of Clinical Epidemiology*.

[31] Pressley JC, Trott C, Tang M, Durkin M, Stern Y (2003). Dementia in community-dwelling elderly patients: a comparison of survey data, Medicare claims, cognitive screening, reported symptoms, and activity limitations. *Journal of Clinical Epidemiology*.

[32] Stuart B, Gruber-Baldini AL, Fahlman C (2005). Medicare cost differences between nursing home patients admitted with and without dementia. *Gerontologist*.

[33] Brauner DJ, Muir JC, Sachs GA (2000). Treating nondementia illnesses in patients with dementia. *Journal of the American Medical Association*.

[34] Kane RL, Bell R, Riegler S, Wilson A, Keeler E (1983). Predicting the outcomes of nursing home patients. *Gerontologist*.

[35] Fleishman R, Rosin A, Tomer A, Schwartz R (1987). Cognitive impairment and the quality of care in long-term care institutions. *Comprehensive Gerontology B*.

[36] (1987). *Diagnostic and Statistical Manual of Mental Disorders, (DSM-III-R)*.

[37] Gruber-Baldini AL, Magaziner J, Zimmerman SI, Kittner S, Hebel JR (1999). MDS assessment of dementia compared to an expert panel. *Gerontologist*.

[38] Menzin J, Lang K, Frtedman M, Neumann P, Cummings JL (1999). The economic cost of Alzheimer’s disease and related dementias to the California medicaid program (“Medi-Cal”) in 1995. *American Journal of Geriatric Psychiatry*.

[39] Folstein MF, Folstein SE, McHugh PR (1975). “Mini mental state”. A practical method for grading the cognitive state of patients for the clinician. *Journal of Psychiatric Research*.

[40] Hartmaier SL, Sloane PD, Guess HA, Koch GG (1994). The MDS Cognition Scale: a valid instrument for identifying and staging nursing home residents with dementia using the Minimum Data Set. *Journal of the American Geriatrics Society*.

[41] Katz S, Ford AB, Moskowitz RW, Jackson BA, Jaffe MW (1963). Studies of illness in the aged. The index of ADL: a standardized measure of biological and psychological function. *Journal of the American Medical Association*.

[42] Fries BE, Schneider DP, Foley WJ, Gavazzi M, Burke R, Cornelius E (1994). Refining a case-mix measure for nursing homes: Resource Utilization Groups (RUG-III). *Medical Care*.

[43] White C (2003). Rehabilitation therapy in skilled nursing facilities: effects of Medicare’s new prospective payment system. *Health Affairs*.

[44] White C, Pizer SD, White AJ (2002). Assessing the RUG-III resident classification system for skilled nursing facilities. *Health Care Financing Review*.

[45] Pope GC, Kautter J, Ellis RP (2004). Risk adjustment of Medicare capitation payments using the CMS-HCC model. *Health Care Financing Review*.

[46] Landis JR, Koch GG (1977). The measurement of observer agreement for categorical data. *Biometrics*.

[47] Huber PJ The behavior of maximum likelihood estimates under non-standard conditions.

[48] White H (1980). A heteroskedasticity-consistent covariance matrix estimator and a direct test for heteroskedasticity. *Econometrica*.

[49] Williams RL (2000). A note on robust variance estimation for cluster-correlated data. *Biometrics*.

[50] Wooldridge JM (2002). *Econometric Analysis of Cross Section and Panel Data*.

[51] (1999). *StatCorp: Stata Statistical Software: Release 6.0*.

[52] Veazie PJ, Manning WG, Kane RL (2003). Improving risk adjustment for Medicare capitated reimbursement using nonlinear models. *Medical Care*.

[53] Centers for Medicare and Medicaid Services http://www.cms.hhs.gov/SNFPPS/09_RUGRefinement.asp.

[54] Consumer Price Index, All Urban Consumers U.S. City Averages. ftp://ftp.bls.gov/pub/special.requests/cpi/cpiai.txt.

[55] Fillit H, Geldmacher DS, Welter RT, Maslow K, Fraser M (2002). Optimizing coding and reimbursement to improve management of Alzheimer’s disease and related dementias. *Journal of the American Geriatrics Society*.

[56] Hood FJ (2001). Medicare’s home health prospective payment system. *Southern Medical Journal*.

[57] http://www.cms.hhs.gov/transmittals/downloads/AB-01-135.pdf.

